# The effect of ulinastatin on sepsis in the rats of adhesion molecules and endothelial function

**DOI:** 10.1186/2197-425X-3-S1-A617

**Published:** 2015-10-01

**Authors:** X Hao, G Cai, C Hu

**Affiliations:** Zhejiang Chinese Medical University, Hangzhou, China; Zhejiang Hospital, Hangzhou, China

## Objectives

To understand the effect of ulinastatin on protecting vascular endothelial cell in sepsis rats and its mechanism.

## Methods

Fifty-two rats were: randomly divided into A(ulinastatin group, n = 26), B (NS, normal saline group, n=26 ). 18 h, 3 h before LPS injection, both groups were pretreated with ulinastatin (100,000 U/kg, dissolved in normal saline of 5 ml)and NS (normal saline, 5 ml), respectively. At the time of 1/2 h, 2 h, 4 h, 12 h, 24 h, 72 h, to measure the level of TNF-α, IL-6, IL-10, VCAM and ICAM-1.

## Results

Compared with group A, the level of TNF-α and IL-6 were increased significantly in group B (P < 0.05); the level of IL-10 were decreased significantly in group B(P < 0.05). The level of VCAM, ICAM-1 in group A were significantly lower than those in group B (P < 0.05).

## Conclusions

Ulinastatin can reduce the mortality of sepsis, its mechanism may be to inhibit the inflammatory response, reduce the release of pro-inflammatory cytokines, promote the expression of anti-inflammatory cytokines and to inhibit the expression of selection, adhesion molecules and angiopoietin receptor, thus it can inhibit the adhesion of inflammatory cells and endothelial cells to improve microvascular permeability and protect the function of endothelial cells.

